# Improved Characterization of Visual Evoked Potentials in Multiple Sclerosis by Topographic Analysis

**DOI:** 10.1007/s10548-013-0318-6

**Published:** 2013-10-02

**Authors:** Martin Hardmeier, Florian Hatz, Yvonne Naegelin, Darren Hight, Christian Schindler, Ludwig Kappos, Margitta Seeck, Christoph M. Michel, Peter Fuhr

**Affiliations:** 1 Department of Neurology, Hospital of the University of Basel, Petersgraben 4, 4031 Basel, Switzerland; 2 Swiss Tropical and Public Health Institute, 4002 Basel, Switzerland; 3 EEG and Epilepsy Unit, Neurology Clinic, University Hospital Geneva, 1211 Geneva, Switzerland; 4 Departments of Basic and Clinical Neurosciences, University of Geneva, 1211 Geneva, Switzerland

**Keywords:** Multiple sclerosis, Visual evoked potentials, Topographic analysis, Quantification, Surrogate marker

## Abstract

In multiple sclerosis (MS), the combination of visual, somatosensory and motor evoked potentials (EP) has been shown to be highly correlated with the Expanded Disability Severity Scale (EDSS) and to predict the disease course. In the present study, we explored whether the significance of the visual EP (VEP) can be improved with multichannel recordings (204 electrodes) and topographic analysis (tVEP). VEPs were analyzed in 83 MS patients (median EDSS 2.0; 52 % with history of optic neuritis; hON) and 47 healthy controls (HC). TVEP components were automatically defined on the basis of spatial similarity between the scalp potential fields (topographic maps) of single subjects’ VEPs and reference maps generated from HC. Non-ambiguous measures of latency, amplitude and configuration were derived from the maps reflecting the P100 component. TVEP was compared to conventional analysis (cVEP) with respect to reliability in HC, validity using descriptors of logistic regression models, and sensitivity derived from receiver operating characteristics curves. In tVEP, reliability tended to be higher for measurement of amplitude (*p* = 0.06). Regression models on diagnosis (MS vs. HC) and hON were more favorable using tVEP- versus cVEP-predictors. Sensitivity was increased in tVEP versus cVEP: 72 % versus 60 % for diagnosis, and 88 % versus 77 % for hON. The advantage of tVEP was most pronounced in pathological VEPs, in which cVEPs were often ambiguous. TVEP is a reliable, valid, and sensitive method of objectively quantifying pathological VEP in particular. In combination with other EP modalities, tVEP may improve the monitoring of disease course in MS.

**Electronic supplementary material:**

The online version of this article (doi:10.1007/s10548-013-0318-6) contains supplementary material, which is available to authorized users.

## Introduction

Prolongation of the P100 latency of the visual evoked potential (VEP) has long been used to detect subclinical demyelinating lesions in multiple sclerosis (MS) localized in the pre- and retro-chiasmal part of the visual pathway through the use of full-field and hemi-field stimulation, respectively (Halliday et al. 1972; Tobimatsu and Celesia 2006). Although no longer explicitly mentioned in the latest revision of the diagnostic criteria for MS (Polman et al. 2011), pathological VEP can provide proof of lesion dissemination in space (Polman et al. 2005; McDonald et al. 2001). Apart from diagnosis, the combination of VEP with motor and somatosensory EP (i.e. multimodal EP) has been shown to be useful for disease monitoring and for defining the long-term prognosis of MS both retrospectively and prospectively (Fuhr et al. 2001; Kallmann et al. 2006; Invernizzi et al. 2011; Schlaeger et al. 2012a, b, 2013).

In order to increase the sensitivity of VEP for subclinical involvement of the optic nerve in MS, a main focus of research lies on advanced stimulation techniques. VEP to low-contrast stimulation have shown more abnormalities than high-contrast stimulation (Kupersmith et al. 1984; Thurtell et al. 2009), and multifocal VEP were reported to detect small or peripheral deficits more sensitively in ON and opposite eyes (Klistorner et al. 2008, 2009; Laron et al. 2009). Furthermore, these two techniques have been recently combined in a pilot study (Frohman et al. 2012). However, the most common way to elicit a robust VEP is still high-contrast full-field pattern stimulation.

For the purpose of disease monitoring, it is also important to quantify pathological VEP with low amplitudes: this may be problematic, particularly with conventional readings. In the present study, we focus on an approach to defining the VEP components independently of amplitude.

Topographic analysis of multichannel evoked potential recordings allows the objective analysis of EPs by defining EP-components based on the spatial distribution of the scalp potential field (i.e. the topographic map) rather than the peak amplitude at a given electrode (Lehmann and Skrandies 1984; Brandeis and Lehmann, 1986; Michel et al. 2001; Murray et al. 2008; Michel and Murray, 2012). In MS, topographic methods have been applied in one small precursor study, in which analysis of 44 healthy controls, 26 MS-patients and 20 patients with other neurological diseases revealed a higher diagnostic sensitivity (72 %) and specificity (100 %) of topographic analysis of VEP (tVEP) compared to conventional waveform analysis (Lascano et al. 2009). In that study, component definition relied on the magnitude of spatial correlation between the measured scalp potential field (i.e. the topographic map) at single time points and reference topographic maps for EP-components derived from a control group. In contrast, conventional analysis depends on the subjective visual identification of the P100 peak at predefined electrodes, and the determination of latency and amplitude at this peak.

In view of the promising results of the report by Lascano et al. (2009), we tested in a larger sample of well-characterized MS patients whether topographic analysis indeed characterizes the P100-component more reliably than conventional readings, especially in pathological VEP. We first determined the reliability of the two methods in a sample of healthy controls measured at baseline and after 1 year. Second, we assessed validity by exploring whether topographic information is useful in distinguishing patients from healthy controls and in detecting prechiasmal demyelination defined as a history of optic neuritis (ON). Third, we determined sensitivity and specificity for diagnosis and detection of a history of ON.

## Materials and Methods

### Subjects

The study was approved by the local ethics committee, and all participants gave written informed consent before inclusion. The baseline sample consisted of 83 MS patients (median age 38.5 years; 80 % female; median Expanded Disability Status Scale (EDSS, Kurtzke 1983) 2.0, range 0–5.5; median disease duration 9.2 years, range 0.3–30.8 years) diagnosed with clinical isolated syndrome (*n* = 5; 6.0 %), relapsing-remitting MS (*n* = 76; 91.6 %) and secondary progressive MS (*n* = 2; 2.4 %) according to Polman et al. (2005). History of optic neuritis (hON) was defined retrospectively by chart review. Clinical standard criteria were used to make the diagnosis: unilateral decline or loss of vision over a period of hours or a few days, pain on eye movement, and decreased perception of color (Balcer 2006). Forty-three patients (52 %) had a positive history of ON. In 28 patients, ON was the first symptom; 19 patients had more than one episode of ON; in three patients, ON had taken place eleven or twelve months prior to the baseline exam. In the hON-group, visual acuity as determined with a Snellen chart was less than 0.8 in 17 eyes of 13 subjects (mean visual acuity: 0.84, SD: 0.24); in the non-hON-group, 5 eyes in 5 subjects had a visual acuity less than 0.8 (mean visual acuity: 0.95, SD: 0.1). All MS patients were examined at prescheduled visits outside a clinical relapse, and at least 4 weeks after corticosteroid treatment for a relapse had been tapered off.

Forty-seven subjects served as healthy controls (HC), defined by an unremarkable personal history, a normal short neurological exam and a corrected visual acuity of 0.8 or better in at least one eye (median age 38.0 years, 75 % female). Thirty-six of these were re-examined after 1 year.

### VEP Recording

Visual EPs were recorded with a 256-channel EEG system (Netstation 200 with HydroCel Geodesic Sensor Net, Electrical Geodesics, Inc., Oregon, USA). The electrode net was placed with Fz, Cz, Oz, and the preauricular points as landmarks. Electrode impedances were kept below 40 kOhm. Recording band-pass was 0.1–100 Hz, sampling frequency 1 kHz, and the vertex was used as the recording reference. Full-field checkerboard stimulation was applied to each eye separately (central fixation; rectangular stimulus field diagonally subtending 10.3° of visual angle; check-size, 30.96′ minutes of arc; 2 × 300 stimuli per eye; 526 ms interstimulus interval; mean luminance 57.5 cd; Michelson contrast, 97 %) according to international guidelines (Celesia and Brigell 1999). Raw data were visually inspected, band-pass filtered (1–30 Hz) and averaged excluding epochs with high amplitude artefacts. Artefact-contaminated electrodes were interpolated using a spherical spline algorithm (Perrin et al. 1989). For topographic analysis, 204 channels were used and re-referenced to their average, leaving out electrodes at the cheeks and the neck.

### Conventional Analysis

Conventional analysis (cVEP) was performed independently by two board-certified neurophysiologists who were blinded to the subjects’ diagnosis. Latency and amplitude (N75- to P100-peak) of the P100 were determined from the waveform recorded at the Oz-Fpz electrode pair for each eye. In 13 VEP, the two readers had differing opinions on the P100 peak, and a consensus was reached.

### Topographic Analysis

Topographic analysis was performed with the free academic software Cartool (Brunet et al. 2011), as has been described previously (Murray et al. 2008; Lascano et al. 2009).

**Fig. 1:**
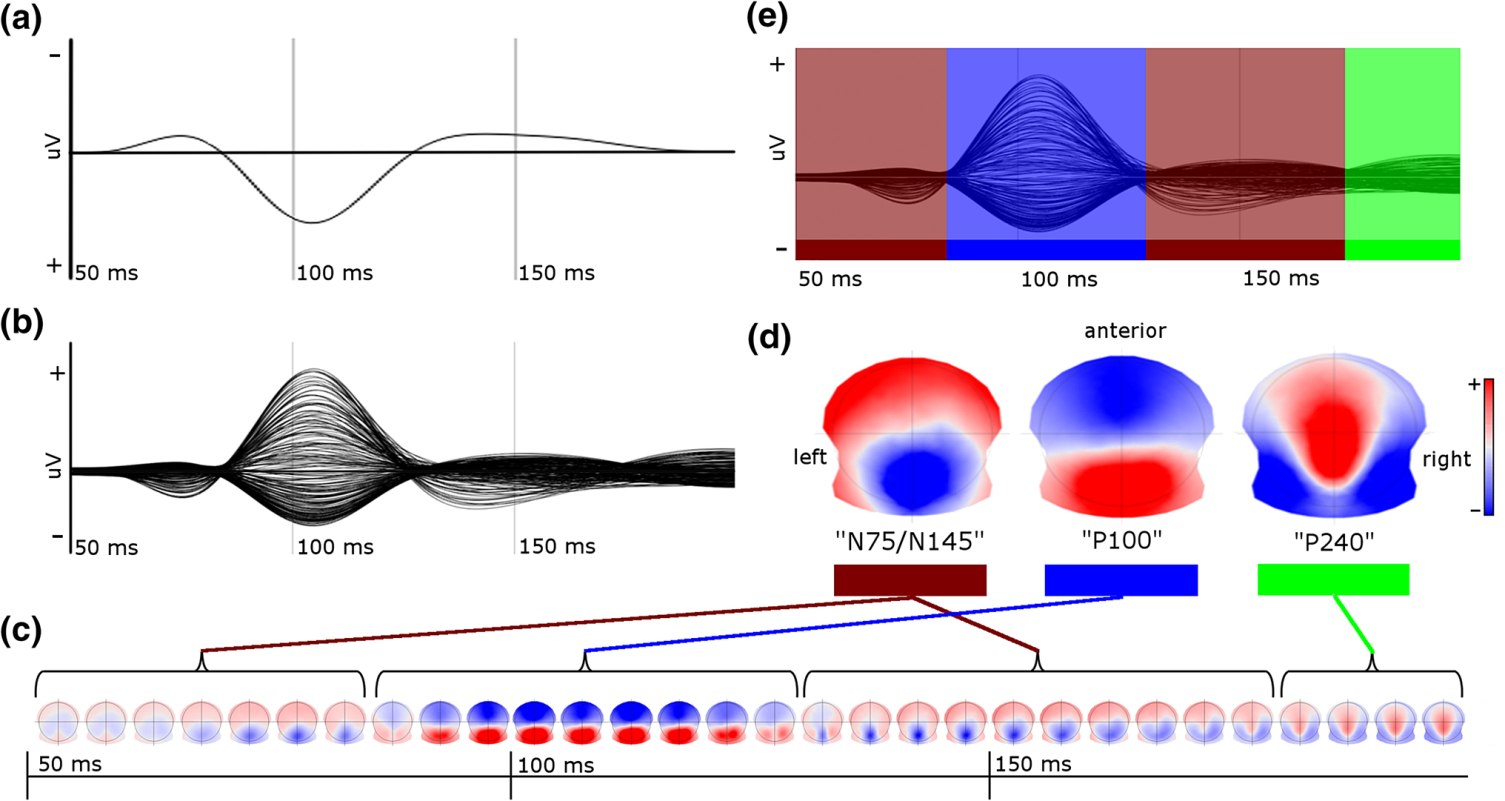
Topographic analysis I: generation of reference maps from healthy controls. **a** Conventional VEP (Oz–Fpz-electrode pair) from the grand mean VEP of all healthy controls. **b** Butterfly plot of the grand mean VEP of all healthy controls derived from 204 electrodes (average reference). **c** Grand mean VEP represented as a time series of topographic maps derived from the butterfly plot: time periods of quasi-stable topographies (“functional microstate”) are flagged by parentheses (for display, each five single topographic maps (=5 ms) are averaged). **d** Reference maps of single EP-components; from left to right, the “N75/N145”-, “P100”- and “P240”-maps are displayed and color-coded. Reference maps are the average of a group of topographic maps with high spatial similarity; N75 and N145 are represented as one reference map because of their overlapping spatial distribution of the electric potential on the scalp. **e** Butterfly plot as in (b) with additional color-coding according to the presence of a component during the time course of the EP, determined by the magnitude of spatial similarity of single topographic maps with one of the reference maps (fitting procedure, see text) (Color figure online)

In contrast to conventional analysis, in which the difference between the electric potentials at two electrodes is measured, topographic analysis relies on the distribution of the electric potential at the scalp (i.e. the topographic map) recorded from a multichannel electrode array. Instead of a voltage time series that forms a waveform for each electrode (Fig. 1a, b), the VEP is represented as a time series of topographic maps, as shown in Fig. 1c for the grand mean VEP of all healthy controls. The time series is characterized by time periods in which topographic maps have a stable and distinct distribution of the electric potential which varies only in intensity. These periods have been called functional microstates (Lehmann and Skrandies 1984; Brandeis and Lehmann 1986; Michel et al. 2001; Murray et al. 2008). Each microstate typically covers the period of a peak in the evoked potential waveform, i.e. what is traditionally called an evoked potential component.

In order to objectively determine the mean topographic map of these components, a k-means cluster analysis can be applied that clusters together all single topographic maps with similar spatial distribution of the potential field regardless of their chronological order. The optimal number of clusters is determined by a cross-validation criterion (Pascual-Marqui et al. 1995; Murray et al. 2008). In our data, this analysis found three cluster maps to be optimal for representation of the traditional EP-components of the grand-mean VEP (Fig. 1d). The cluster algorithm did not distinguish between the N75 and N145 components, as the single topographic maps in the time windows 50–87 ms and 135–180 ms were spatially very similar; therefore, they are represented as a single mean topographic map. In order to define the time at which each component is present, the spatial correlation between the mean topographic maps and each single topographic map of the time series is calculated. Subsequently, each point in time is defined as belonging to the component to which the magnitude of correlation is highest. This fitting procedure revealed, as expected, that the three mean topographic maps represent the periods traditionally labeled N75, P100, N145, and P240 (Fig. 1e). In subsequent analysis, these mean topographic maps will be used as reference maps and referred to as the “N75/N145”-, “P100”-, and “P240”-maps (Fig. 1d).

**Fig. 2:**
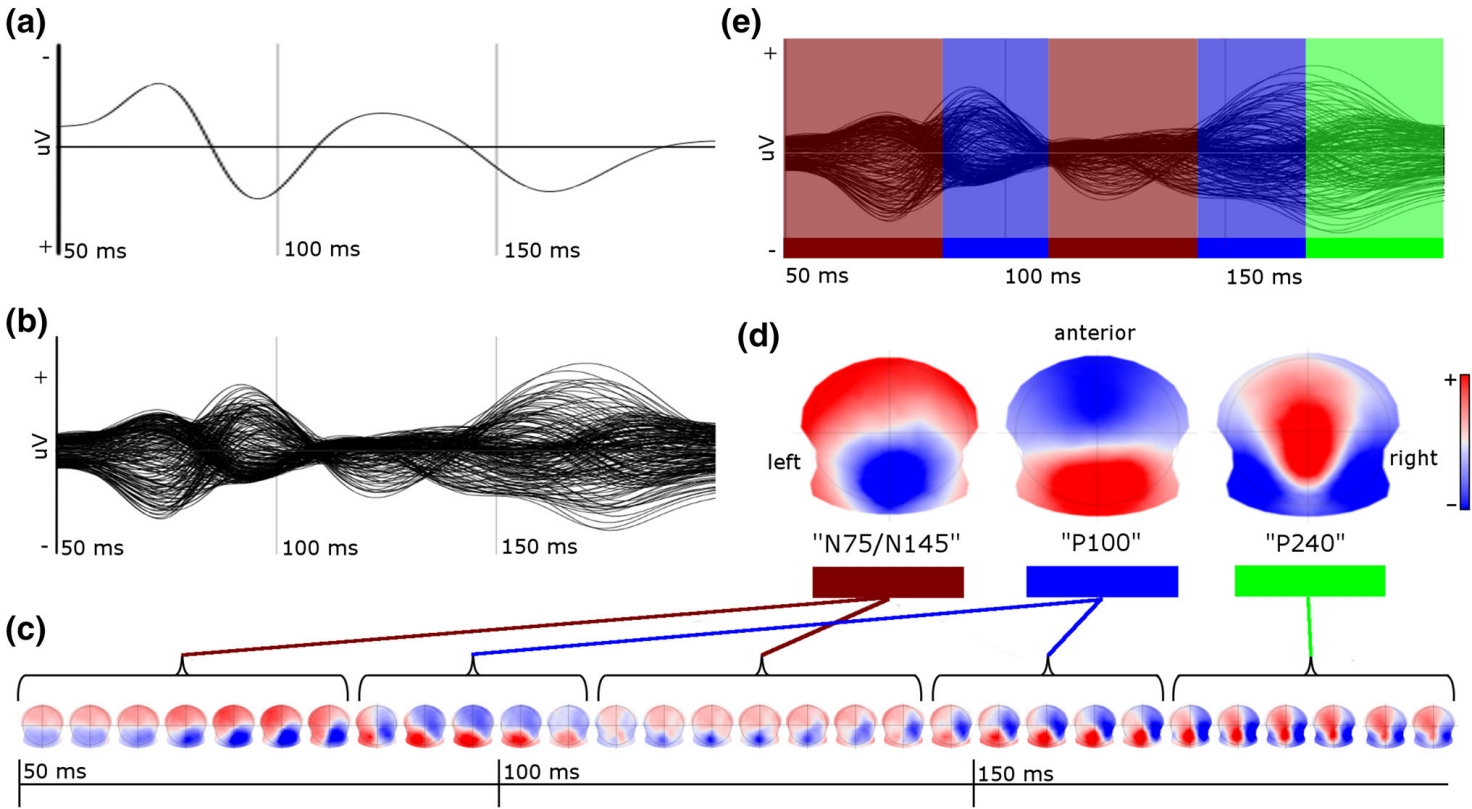
Topographic analysis II: automatic definition of EP components in an individual VEP. **a** Conventional VEP (Oz–Fpz-electrode pair) in a healthy control. **b** Butterfly plot of same subject derived from 204 electrodes (average reference). **c** VEP represented as a time series of topographic maps derived from the butterfly plot. (For display, five single topographic maps (=5 ms) are averaged). Marked asymmetry is seen despite monocular full-field stimulation. **d** Reference maps of single EP components derived from all healthy controls (see Fig. 1d). **e** Butterfly plot as in (b) with additional color-coding according to the presence of a component during the time course of the EP, determined by the magnitude of spatial similarity of single topographic maps with one of the reference maps (fitting procedure, see text) (Color figure online)

**Fig. 3:**
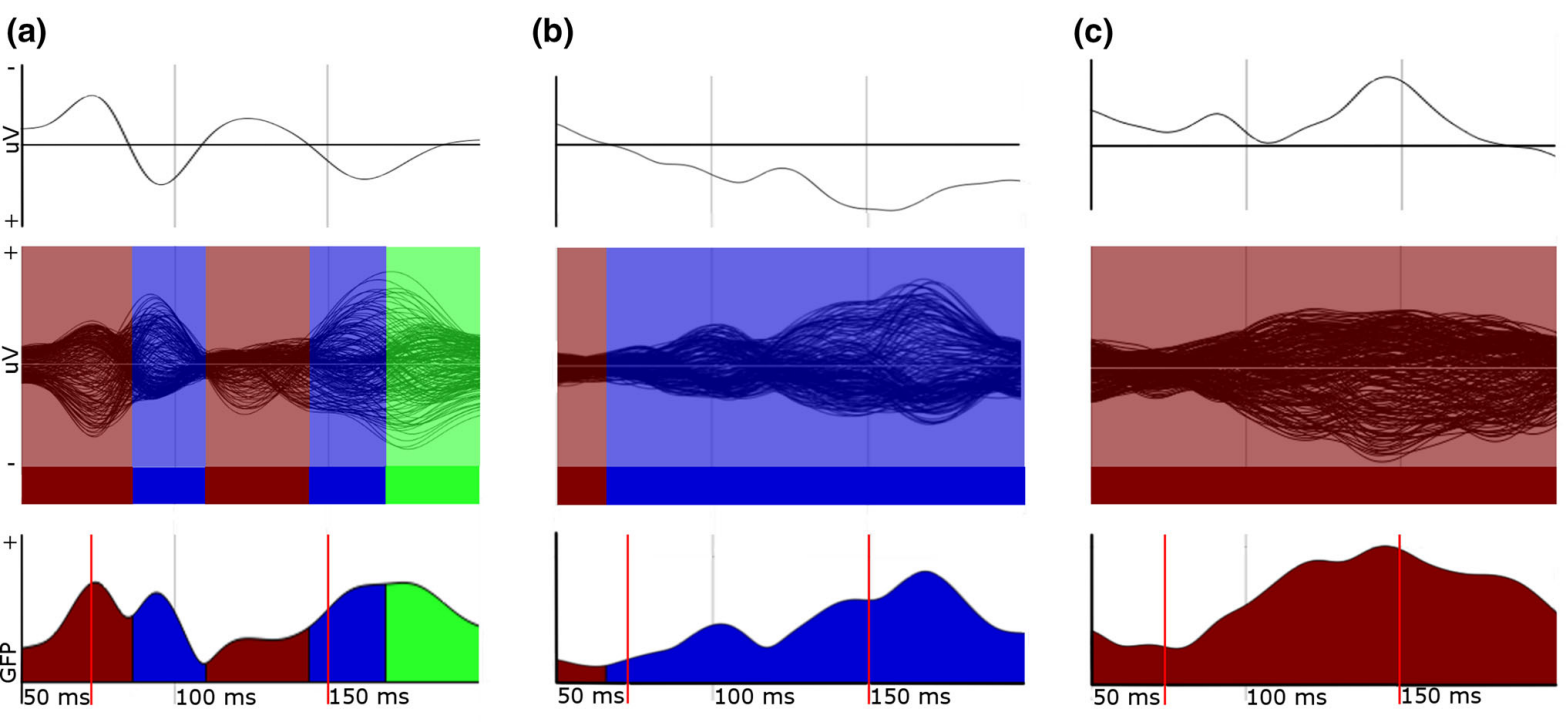
Examples of single VEPs in a healthy subject (**a**) and two patients (**b**, **c**); *upper panel*: conventional VEP; *middle panel*: butterfly plots with topographically defined, color-coded EP components; *lower panel*: corresponding time course of GFP with respective color-coding. (*GFP* global field power, *uV* microVolt; *red lines*: time window for quantitative analysis). **a** Same healthy subjects as in Fig. 2: in addition to conventional waveform and butterfly plot with color-coded EP-components (see Fig. 2), the time course of the GFP and the time window for analysis is shown (*lower panel*). A wider time window would have falsely given the late peak as the latency of the P100 component. **b** MS patient with positive history of ON, visual acuity 0.5, EDSS 4.0: conventional waveform shows a small and a high positive peak at 95 and 160 ms, latency and amplitude measurement is ambiguous; the color-coding of the butterfly plot and the time course of GFP reflect the fact that spatial similarity of topographic maps (see Fig. S1) is highest to the “P100”-reference; for analysis, the latency at the end of the time window is used (tLat = 150 ms; replacement procedure 3). **c** MS patient with positive history of ON, visual acuity 0.5, EDSS 2.0: conventional waveform shows a shallow peak at 103 ms; the color-coding of the butterfly plot and the time course of GFP reflect the fact that spatial similarity of topographic maps (see Fig. S2) is highest to the “N75”-reference; for analysis, the most pathological values of latency, amplitude and configuration (tLat, tAmp, tAUC and tFit) measured in the sample are used (replacement procedure 2) (Color figure online)

Figure 2 displays the fitting procedure applied to an individual VEP of a healthy subject. From the butterfly plot (Fig. 2b) the individual time course of topographic maps is derived (Fig. 2c), and each time point of the butterfly plot is color-coded (Fig. 2e) according to the magnitude of the spatial correlation of the corresponding topographic map to one of the reference maps (Fig. 2d). In order to quantify the field strength of the VEP at each time point, the global field power (GFP) was used (Lehmann and Skrandies 1980). GFP is defined as the standard deviation of the mean amplitude over all electrodes at a single time point. Figure 3a shows in the same healthy subject as in Fig. 2 the GFP time course (lower panel) derived from the butterfly plot (middle panel) with corresponding color-coding. Supplemental figures (Fig. S1 and S2) depict the fitting procedure including the time series of topographic maps for the two MS subjects shown in Fig. 3b, c.

For analysis, the following parameters were used from the topographically defined P100 component: topographic amplitude (tAmp) given as the maximal GFP, topographic latency (tLat) given as the time point of maximal GFP, “configuration” (tFit) given as the maximal value of spatial correlation to the reference map, and the mean amplitude (tAUC) given as the total sum of GFP while the P100 component was present, corresponding to the area under the component curve of the GFP time course (Fig. 3, lower panel). In very pathological VEP, in which all single topographic maps show a higher spatial correlation to the “N75/N145-” or “P240”-map than to the “P100”-map, the fitting procedure only yields these components, but no P100 component (Fig. 3c and Fig. S2). To include these very pathological VEP in the statistical analysis, as well as conventional VEP in which no P100 peak could be defined, values were replaced by the most pathological measured values of the sample, as described below.

The time window for detection of the P100 component was restricted to 70–150 ms in order not to quantify late components with a P100 topography and high GFP as P100 latency and P100 amplitude, despite a clear but lower peak of the P100 component with normal latency, as shown for a healthy control in Fig. 3a. Consequently, in MS cases with very prolonged latencies, the true peak lies outside this time window, and thus the end of the time window is recognized as the peak of the P100 component (Fig. 3b and Fig. S1).

### Data Pre-processing

The distributions of all calculated values from conventional and topographic analysis were tested for normality using q–q-plots and a Kolmogorov–Smirnov-test, and log-transformed when necessary. Control subjects were then used as the reference sample for z-transformation, and the mean z-value of each subject’s left and right VEP was used for statistical analysis.

In order to analyze all subjects (*n* = 83 patients, *n* = 47 HC), it was necessary to replace the VEP values of eyes in which no valid conventional or topographic P100 could be determined. Three replacement procedures were employed. In VEPs of eyes with pathology other than MS, or visual acuity below 0.8 in control subjects, values were replaced by the values of the VEP from the subject’s opposite eye (procedure 1). In VEPs in which no P100 peak could be visually determined or no P100 component could be defined topographically (Fig. 3c and Fig. S2), the most pathologically measured values of the sample were used (procedure 2), as suggested previously (Fuhr et al. 2001; Schlaeger et al. 2012b). The same replacements were done in VEP from eyes with visual acuity of 0.2 or less due to ON, as recordings may not be reliable because of poor fixation. In tVEP, in which the true P100 peak lay outside the predefined time window (Fig. 3b and Fig. S1), the end of the time window (150 ms) was taken as the latency (procedure 3); when the tVEP peak was at the very beginning of the time window (<80 ms), it was also replaced with the maximal topographic latency (150 ms) as such a non-physiologic early peak was considered to reflect severe pathology.

Table 1 gives the number of subjects and VEPs, reasons for replacements, and replacement procedures. In topographic analysis, 31 VEPs in 25 patients were affected; 19 VEPs in 17 patients had to be replaced and 12 VEPs in 10 patients had the true peak outside or at the very beginning of the pre-defined time window (two subjects are counted twice because of a replacement in one eye and a peak outside the time window in the other eye). In conventional analysis, 14 VEPs in 12 patients were replaced. In the 2 VEPs from the 2 patients’ eyes with non-MS-pathology (strabismic and congenital amblyopia), the values of the same subject’s opposite eye were used. In 8 VEPs from 6 patients without discernible P100 peak and in the 4 VEPs of the 4 patients’ eyes with visual acuity <=0.2, the most pathological measured conventional values were used. In healthy controls, 5 VEPs in 5 subjects (baseline) and 2 VEPs in 2 subjects (year 1) were replaced by the respective conventional and topographic values of the same subject’s opposite eye, as the visual acuity was less than 0.8 at the replaced side because of an uncorrected refractive error.

**Table 1:** Number of MS-patients and VEPs with replacement of non-valid values for topographic analysis (see text for conventional analysis)

Subjects^a^	VEPs	Reason	Replacement by
2	2	Non-MS pathology^b^	tLat, tAmp, tAUC, tFit of VEP of same subject’s opposite eye
11 4	13 4	No P100 component visual acuity <= 0.2	Most pathological tLat, tAmp, tAUC, tFit measured in the sample^c^
9 1	11 1	True peak outside time window non-physiological early peak (79 ms)	tLat = end of time window = 150 ms^c^

^a^Two subjects are counted twice because different reasons for replacements in VEP from right and left eye

^b^one strabismic, one congenital amblyopia

^c^see Fig. 3b, c

To estimate the effect of replacements, sensitivity analyses were performed in the 58 patients and 42 healthy controls without replacements.

## Statistics

The R-project software package (Version 2.12.1) and SPSS (SPSS IBM Statistics, version 20.0) were used for statistical analyses.

### Test–Retest-Reliability

In healthy controls, the intraclass correlation coefficient between corresponding baseline and year 1 values was calculated for each conventional and topographic measure. The standard deviation of the difference between baseline and year 1 was used to describe variability and compared between the two methods by Pitman’s test (Pitman 1939; Howell 1997). Pitman’s test is based on the idea that, if there is no significant difference between two methods, there should be no significant correlation between the sum and the difference of the differences between baseline and year 1 measured with method A or method B.

### Validity

In order to compare conventional and topographic measures as predictors of diagnosis (MS vs. healthy control), descriptors of logistic regression models (stepwise backwards procedure; log-likelihood-ratio; *p* in = 0.1, *p* out = 0.11) were used. Model comparisons were based on the amount of explained variability adjusted for number of predictors (adjusted pseudo-R2) and the Bayesian information criterion (BIC). The BIC reduces the risk of over-fitting by penalizing the complexity of the model, and thus is a more meaningful model descriptor compared to the adjusted pseudo-R2. The same analysis was repeated within patients using “history of optic neuritis” instead of diagnosis as the dependent variable in the logistic regression.

Using the z-transformed values of the VEPs of the subjects’ left and right eyes in mixed regression models with the subject as random factor, instead of the mean z-values of the VEPs of the two eyes, yielded similar results to the described approach (data not shown). As using the mean z-values of the VEPs of the two eyes is a simpler way to account for the fact that the VEPs from a subject’s left and right eye are not independent observations, this method was preferred.

### Sensitivity and Specificity

Receiver operating characteristics (ROC) curves were calculated for all models, and sensitivity and specificity were determined at the cut-points of the ROC-curve which maximizes the sum of sensitivity and specificity (index of Youden YI).

## Results

### Reliability

In healthy controls, the intraclass correlation coefficient between baseline- and year 1-values was highest for tLat (*r* = 0.95) and cLat (*r* = 0.94), followed by tAmp (*r* = 0.81), tAUC (*r* = 0.75), and cAmp (*r* = 0.73), and was lowest for tFit (*r* = 0.67). The variability of longitudinal change, expressed as its standard deviation (SD), showed no significant difference between topographic and conventional latency (tLat: mean change = −0.05, SD = 0.34; cLat: mean change = −0.05, SD = 0.37; *p* = 0.095; absolute mean change without z-transformation: tLat = 1.74 ms, SD = 1.44; cLat = 1.72 ms, SD = 1.59), and there was only a statistically insignificant trend toward lower variability (16 %) in topographic as compared to conventional amplitude (tAmp: mean change = −0.10; SD = 0.57; cAmp: mean change = −0.07, SD = 0.68; *p* = 0.059).

### Validity

The explained variability, expressed as the adjusted pseudo-R2 and the Bayesian information criterion (BIC) as descriptors of the logistic regression models, is shown in Table 2. The models for the outcome diagnosis explained 28 % of the variability (BIC: 130) with a combination of tLat (*p* < 0.001) and tFit (*p* < 0.05), and 25 % (BIC: 131) with cLat (*p* < 0.001). The models for the outcome history of ON explained 35 % of the variability (BIC: 82) with a combination of tLat (*p* < 0.001) and tAUC (*p* = 0.07), and 26 % (BIC: 91) with a combination of cLat (*p* < 0.01) and cAmp (*p* < 0.05). In the models on history of ON, the BIC was consistently lower with topographic VEP measures than in the models with conventional measures; this implies that the former had a higher information content. When cases with replacements were omitted in sensitivity analyses, conventional measures had the lowest BIC; in the model on “diagnosis”, cLat (*p* < 0.001) explained 14 % of the variability (BIC: 121) and a combination of tLat (*p* < 0.001 and tFit (*p* < 0.05) explained 16 % of the variability (BIC: 123); in the model on “history of ON”, a combination of cLat (*p* < 0.01) and cAmp (*p* < 0.05) explained 26 % of the variability (BIC: 64), and tLat (*p* < 0.001) explained 21 % of the variability (BIC: 65).

**Table 2:** Descriptors of logistic regression models on “diagnosis” (MS vs. HC) and “history of optic neuritis” for conventional (c) and topographic (t) predictors (apR2: adjusted pseudo-R2; BIC: Bayesian information criterion)

	“Diagnosis”		“History of optic neuritis”	
	apR2	BIC	apR2	BIC
cLat	**0.25**	**131**	0.21	94
cLat + cAmp	0.25	136	**0.26**	**91**
tLat	0.26	131	**0.32**	**81**
tLat + tFit	**0.28**	**130**	0.32	85
tLat + tAUC	0.26	134	**0.35**	**82**
tLat + tAmp	0.26	133	0.34	83

*Bold* models with lowest BIC and/or highest apR2

### Sensitivity and Specificity

The sensitivity and specificity of conventional and topographic measures in predicting diagnosis and history of ON at the point maximizing the index of Youden (YI) are given in Table 3. Diagnosis was best predicted by a combination of tLat and tFit, which was more sensitive but somewhat less specific than the combination of cLat and cAmp (sensitivity: 72 vs. 61, specificity: 87 vs. 92; YI: 0.60 vs. 0.53). History of ON was best predicted by a combination of tLat and tAUC, with a clear increase in sensitivity compared to the model with cLat and cAmp (sens: 88 vs. 77; spec: 83 vs. 85; YI: 0.71 vs. 0.62). Using only latency as a predictor for history of ON increased specificity at the cost of sensitivity, with tLat being superior to cLat (sens: 79 vs. 70; spec: 90 vs. 90; YI: 0.69 vs. 0.60).

**Table 3:** Comparison of sensitivity and specificity of conventional (c) and topographic (t) measures in predicting diagnosis (MS vs. HC) and history of optic neuritis

	“Diagnosis”		“History of optic neuritis”	
	Sensitivity	Specificity	Sensitivity	Specificity
cLat	60	89	70	90
cLat + cAmp	61	92	77	85
tLat	60	91	79	90
tLat + tFit	**72**	**87**	79	90
tLat + tAUC	68	89	**88**	**83**
tLat + tAmp	75	75	**84**	**88**

*Bold* values at highest index of Youden (=maximal sum of sensitivity and specificity)

## Discussion

In the present study, topographic analysis of the P100 component of the VEP is compared to conventional readings in a large group of well-characterized MS patients and healthy controls. A trend for higher test–retest reliability is observed for the topographic assessment of amplitude measures in healthy controls. Diagnostic yield for MS is higher and prediction of a history of optic neuritis is better with the topographic method. However, the conventional method performs equally well in discriminating between the two groups and in predicting a history of optic neuritis when the most pathological VEPs are excluded. Thus, the advantage of topographic analysis lies in the quantification of difficult VEPs, in which conventional waveforms are frequently ambiguous and no conclusion can be made. However, even in the more straightforward cases of normally configured VEP, the fact that the topographic analysis is automatic and does not rely on subjective decisions of experienced investigators can still be an advantage.

For monitoring the disease course, the use of only the most robust EP components has been recommended (Comi et al. 1999) and has been found useful (Fuhr et al. 2001; Schlaeger et al. 2012a, b, 2013). In the present study, the P100 latency shows highest test–retest reliability in the same range as reported previously (Meienberg et al. 1979; Thomae et al. 2010) and is the main factor in predicting diagnostic group and history of ON.

However, diagnostic sensitivity is increased by considering topographic fit as an additional factor. Topographic fit represents the maximal spatial correlation of each subject’s time series of topographic maps to the reference maps derived from healthy controls. Low spatial correlation is expected in asymmetries or distortion of the field distribution. In conventional recordings, marked amplitude asymmetries between lateral recording electrodes after full-field stimulation can be a sign of a retrochiasmal lesion (Blumhardt and Halliday 1978). Unfortunately, amplitude asymmetries are quite insensitive: even in subjects with gross hemispheric lesions and hemianopsia, amplitude asymmetries to full-field stimulation are still within normal limits in 45 % of patients (Blumhardt et al. 1982), as the physiological variability of amplitude asymmetries is high. However, the influence of retrochiasmal lesions may be one possible explanation for the increased diagnostic sensitivity when topographic fit is used, because spatial correlation does not depend on amplitudes but amplitude asymmetries may alter the scalp field distribution of the potential.

The inclusion of amplitude measures markedly increases the sensitivity of detection of a prechiasmal lesion defined as a positive history of ON, with a clear advantage for topographic measures. This observation suggests that amplitude may carry complementary information to latency. This suggestion is supported by the fact that in MS, VEP amplitude but not latency is associated with reduced thickness of the retinal nerve fibre layer and with decreased macular volume (Trip et al. 2005), as well as with optic nerve atrophy (Trip et al. 2006).

One reason why amplitude measures were found to be less informative than latency in previous longitudinal studies (Brusa et al. 1999, 2001) may be that they are less reliable, so that statistical inferences are more difficult. Conventional amplitude assessment depends on the P100 and N75 peaks, which may not be maximal at predefined electrode positions in individuals. Furthermore, the N75 is more variable than the P100 (Meienberg et al. 1979; Thomae et al. 2010). One way to make amplitude measurement more reliable is to optimize stimulation by using multifocal VEP, in which the central and peripheral visual field are stimulated simultaneously (Klistorner et al. 2008, Laron et al. 2009). Using this technique, the amplitude in the non-affected eye was shown to be lower in patients at high risk for multiple sclerosis than in those with a low risk twelve months after a first ON (Klistorner et al. 2009). In contrast, amplitude measurement in topographic analysis is optimized by the use of the global field power, which reflects the field strength measured over all electrodes, and by relying only on the P100 component, thus eliminating both electrode position and the N75 as sources of variability. Combining an optimized stimulation technique with an optimized measurement technique might further reduce the variability of the VEP amplitude. However, the potential clinical benefit of an improved assessment of amplitude and configuration regarding future functional impairment remains to be determined in longitudinal studies.

In the present study, the findings of Lascano et al. (2009) regarding the validity of topographic analysis are confirmed and extended in a larger sample of MS patients and with a presumably wider range of pathologic abnormalities. In both studies, the sensitivity for a diagnosis of MS is found to be higher for topographic than for conventional measures (72 % vs. 60 % present study; 72 % vs. 56 % Lascano et al. 2009). Furthermore, the present study reveals an advantageous high sensitivity (88 %) and specificity (83 %) of topographic measures for the detection of clinical and subclinical prechiasmal changes.

Ill-defined, pathological VEPs generally pose problems for analysis, as the definition of the P100 component is often ambiguous in these cases. Topographic component definition is advantageous here, as it relies on the distribution of the electric potential on the scalp, rather than on the peak height, and automatically determines whether a P100 component is present. However, VEPs of eyes with visual acuity of 0.2 or less had to be excluded from automatic component detection, as noise can resemble a P100 field distribution in such cases. A specific limitation of tVEP is the use of a fixed time window, which reduces the dynamic range of the method. The use of a time window of 70–150 ms allowed the measurement of values in most MS patients in the present study; still, 6.6 % of the VEP had a peak outside this time window. However, with a larger time window, late components with a predominant P100 field distribution and high peaks would have been mistaken for the P100 latency even in healthy controls. A smaller time window (89–133 ms), as used in the study of Lascano et al. (2009), would have further reduced the dynamic range. In our data, 12.4 % of VEPs would have had the peak outside the given window. However, the significance of such a reduced dynamic range has to be determined longitudinally. A further limitation of the method is the laborious pre-processing that it currently requires.

As neuro-degeneration in MS is only incompletely understood and not well targeted by the available therapeutic options, suitable biomarkers still need to be developed (Ziemann et al. 2011). The non-systematic involvement of different functional systems requires the combination of different EP modalities for an adequate characterization of the multifocal disease process. Still, each modality should add sensitively reliable information. Thus, advanced VEP techniques may increase the known prognostic value of multimodal evoked potentials (Fuhr et al. 2001; Kallmann et al. 2006; Schlaeger et al. 2012a, b, 2013). Furthermore, cognitive symptoms may be quantified by measurement of the P300 in oddball tasks (Whelan et al. 2010; Kiiski et al. 2011) or by measures of neuronal coordination (Leocani et al. 2000; Tecchio et al. 2008; Hardmeier et al. 2012). Beyond evoked potentials, combination of different methods may turn out to be the most successful way to capture the heterogeneity of the disease (Ziemann et al. 2011).

## Conclusions

This study demonstrates the reliability, validity and sensitivity of an automated detection of VEP and suggests a role for multichannel recording and topographic analysis of the VEP in the characterization of the disease course of MS, which requires maximal objectivity in the assessment of as many parameters of CNS function as possible. Longitudinal studies are warranted to address this question further.

## Electronic supplementary material

Below is the link to the electronic supplementary material.

Supplementary material 1 (PDF 133 kb)

Supplementary material 2 (PDF 138 kb)
