# Supplementary material for: Improved Characterization of Visual Evoked Potentials in Multiple Sclerosis by Topographic Analysis
Source: Brain Topogr. 2013 Oct 2;27(2):318–27. doi: 10.1007/s10548-013-0318-6 (PMC3921459; doi:10.1007/s10548-013-0318-6)
Supplement: Supplementary file 1 — Supplementary material 1 (PDF 133 kb) [file 10548_2013_318_MOESM1_ESM.pdf]

Fig. S1

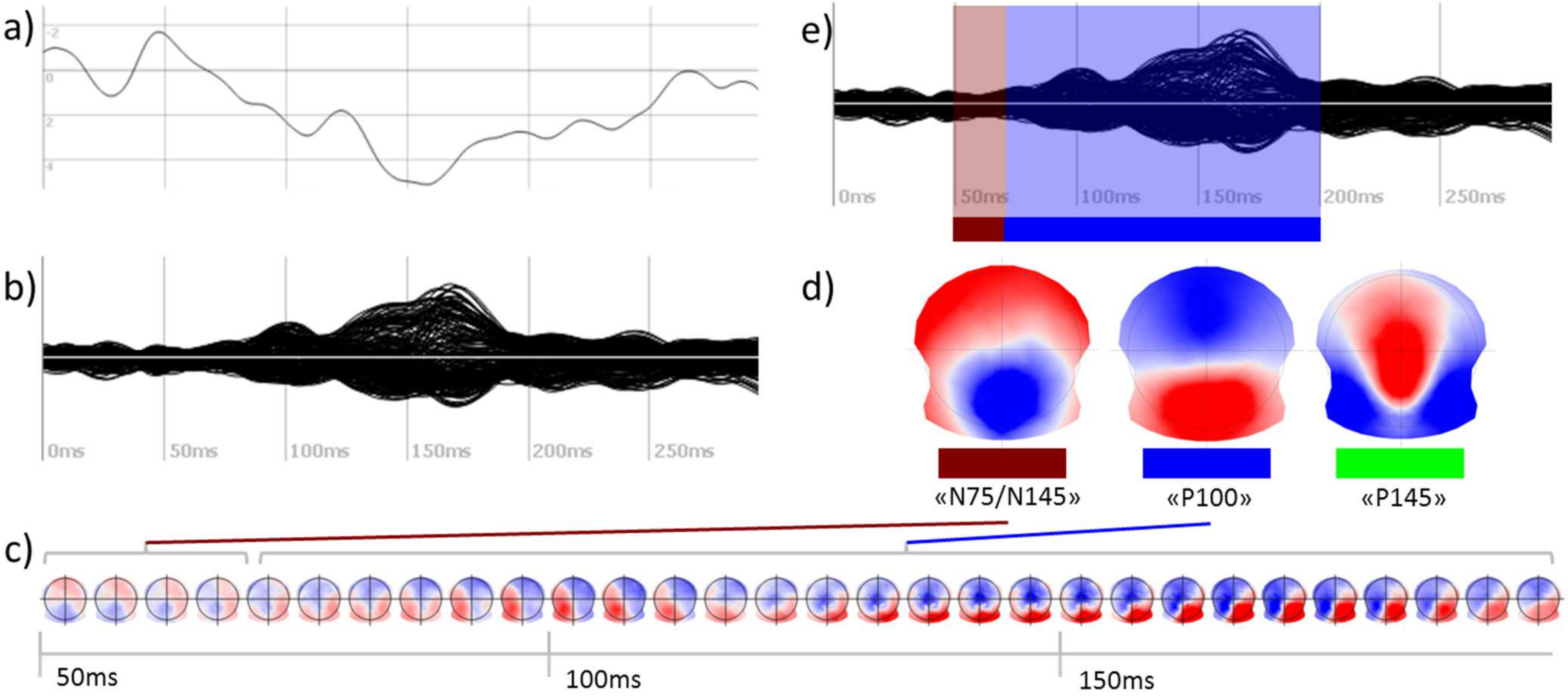

MS-patient with positive history of ON, visual acuity 0.5, EDSS 4.0 (same subject as in Fig. 3b)

a) Conventional VEP (Oz – Fpz electrodes)
b) Butterfly plot (204 electrodes, average reference)
c) Time course of topographic maps derived from the butterfly plot
d) Reference maps for the different EP-components derived from the grand mean VEP of healthy controls (see Fig. 1d)
e) Butterfly plot with color-coding according to the spatial similarity of individual topographic maps to one of the reference maps
