# Supplementary material for: Improved Characterization of Visual Evoked Potentials in Multiple Sclerosis by Topographic Analysis
Source: Brain Topogr. 2013 Oct 2;27(2):318–27. doi: 10.1007/s10548-013-0318-6 (PMC3921459; doi:10.1007/s10548-013-0318-6)
Supplement: Supplementary file 2 — Supplementary material 2 (PDF 138 kb) [file 10548_2013_318_MOESM2_ESM.pdf]

Fig. S2

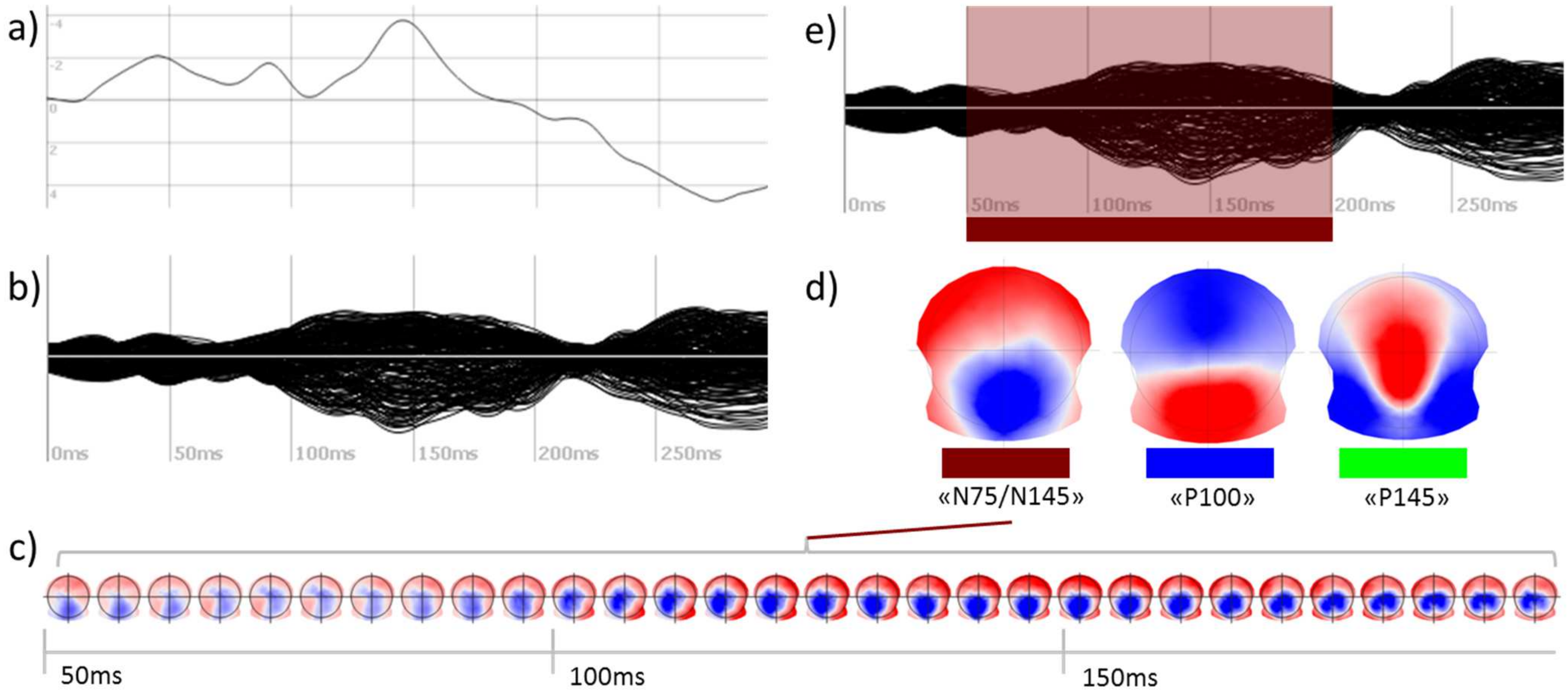

MS patient with positive history of ON, visual acuity 0.5, EDSS 2.0 (same subject as in Fig. 3c)

a) Conventional VEP (Oz – Fpz electrodes)

b) Butterfly plot (204 electrodes, average reference)

c) Time course of topographic maps derived from the butterfly plot

d) Reference maps for the different EP-components derived from the grand mean VEP of healthy controls (see Fig. 1d)

e) Butterfly plot with color-coding according to the spatial similarity of individual topographic maps to one of the reference maps
